# Childhood Asthma-Management Practices in Rural Nigeria: Exploring the Knowledge, Attitude, and Practice of Caregivers in Oyo State

**DOI:** 10.3390/children10061043

**Published:** 2023-06-11

**Authors:** Oyindamola Akinso, Atin Adhikari, Jingjing Yin, Joanne Chopak-Foss, Gulzar Shah

**Affiliations:** 1 Department of Public Health, Wingate University, Wingate, NC 28174, USA; 2 Department of Health Policy and Community Health, Jiann-Ping Hsu College of Public Health, Georgia Southern University, Statesboro, GA 30460, USA; jchopak@georgiasouthern.edu (J.C.-F.); gshah@georgiasouthern.edu (G.S.); 3 Department of Biostatistics, Epidemiology, and Environmental Health Sciences, Jiann-Ping Hsu College of Public Health, Georgia Southern University, Statesboro, GA 30460, USA; aadhikari@georgiasouthern.edu (A.A.); jyin@georgiasouthern.edu (J.Y.)

**Keywords:** asthma management behavior, pediatric asthma, childhood asthma management, caregivers, wheezing, asthma triggers, inhaler, rural communities, Nigeria

## Abstract

**Background:** Caregivers of asthmatic children have a poor knowledge of proper asthma-management practices in Nigeria. This study examined the knowledge, attitudes, and practice behaviors of caregivers in the management of asthma in children under 5 years of age in Oyo State, Nigeria. **Methods:** While a mixed method was used in the original research, this brief describes the quantitative method used in this study to evaluate caregivers’ asthma-management practices. A 55-item questionnaire on childhood asthma knowledge, attitude, and practice was administered during child welfare-clinic visits to 118 caregivers. Data were analyzed using the IBM SPSS Version 25.0. Statistical significance was set at *p* < 0.05 and 95% CI. **Result:** More than 70% of caregivers knew that asthma is associated with airway inflammation and about 90% knew that flu infections triggered asthma attacks in their children. Caregivers with a higher income (OR = 3.0; 95% CI = 1.558–5.778; *p* = 0.001) were 3.0 times more likely to practice proper asthma-care behavior than those with a lesser income. **Conclusions:** Childhood asthma remains underdiagnosed and undertreated in Nigeria. An optimal public health approach is needed to identify and target underserved communities that suffer poorer asthma outcomes and to improve caregivers’ knowledge and practices of asthma management.

## 1. Introduction

Asthma is a significant global health problem and a leading cause of respiratory disorders among children around the world [1]. In Nigeria, the prevalence of asthma remains unknown with certainty caused by insufficient literature on the burden of asthma [2]. Nevertheless, there are little data to estimate the prevalence burden of asthma in Nigeria; prevalence rates have been described as ranging from 5.1% to 14.3% in children and 7–18% in the general population of adults between the ages of 15–35 years [3,4].

Childhood asthma not only affects symptomatic children but also their caregivers because children depend on them for care. Caregivers experience challenges in the pre-identification and management of early asthma symptoms and potential triggers of asthma exacerbation, sometimes not knowing how and when to practice appropriate asthma-preventive measures and seek emergency care or being able to differentiate asthma symptoms from other health conditions [5]. Reports in the literature indicate that caregivers residing in rural communities are confronted with the profound hardship of poverty and are unable to afford quality asthma care for their children [6,7]. Several factors contribute to the poor management of childhood asthma in rural Nigeria. Some of these factors are related to poor standards and access to quality primary care, such as the lack of proper health infrastructure and a shortage in the rural health workforce [6,7]. Similarly, low educational attainment, poor economic background, cultural and religious beliefs, attitudes toward chronic illnesses, and psychological stressors have also been shown to affect the asthma-management practice behaviors of caregivers [8]. However, the extent to which some or a combination of these factors impact childhood asthma-management practices has not been investigated. In developing countries such as Nigeria, there is a paucity of reports on childhood asthma-management practices by caregivers in resource-poor communities. To address these gaps in knowledge, this study was conducted to assess the knowledge, attitude, and practice among caregivers of children with asthma and identify the factors associated with their knowledge and beliefs of asthma-management practices.

## 2. Materials and Methods

### 2.1. Study Design and Sample Population

Although this study employed an explanatory sequential mixed-method design and a multistage sampling technique, the focus of this paper is the quantitative methodology used in this research. The study was conducted in ten healthcare facilities within three local government areas in Oyo State within the Ibadan Metropolitan area. Participants were selected using a multistage sampling of caregivers of children with wheezing or asthma episodes in the last 12 months from May to July 2020. The sampling process for this study included three stages (i.e., two-stage random sampling of health facilities and convenience sampling of research subjects). 

A clustered sampling was used in the first stage of sampling for the selection of local government areas (LGAs). This was followed by a simple random sample of clusters (LGAs) which yielded 3 LGAs from the 11 LGAs in the Ibadan Metropolitan area. Of the 109 health facilities within the 3 LGAs, 9 primary healthcare facilities and 1 state hospital were selected at random. Within the 10 health facilities, participants were selected based on availability (convenience sampling). Recruitment of participants was conducted on child welfare-clinic days at the health facilities. The study included parents and guardians of pediatric patients with asthma aged between 1 and 5 years. In addition, caregivers of children with wheezing or asthma episodes in the last 12 months were also identified from the weekly patient registration log and invited by the medical staff to participate in this study. Study participants who did not meet these criteria were excluded from the study. *The term “parents” in this study includes the main caregivers of children in the home. Voluntary informed consent to participate in this research was obtained from study participants. Approval to conduct this study was obtained from the Institutional Review Board (IRB) of Georgia Southern University, and the Oyo State chapter of the National Health Research Ethics Committee Nigeria (NHREC).

### 2.2. Study Procedure

At the start of the interview, the interviewer screened for suitability and enrolled those who met the eligibility criteria. Face-to-face interviews were carried out with 118 caregivers from May to July 2020. Enrolled participants gave both written and verbal consent, respectively. 

***The questionnaire*:** A research team member administered a 55-item questionnaire. The knowledge domain was adapted from the 17-item asthma knowledge questionnaire (AKQ). The AKQ is a disease-specific questionnaire developed and validated by Martínez and Sossa, with questions on knowledge about symptoms, triggers, and interventions [9]. The attitude and practice domain were adapted from the Child Asthma Risk Assessment Tool (CARAT), a 57-item questionnaire developed by the National Cooperative Inner-City Asthma Study to determine the impact of primary care clinic-based interventions on symptomatic days (http://Carat.asthmarisk.org/, (accessed on 15 November 2018)) [10]. The modified version of this instrument has been used in another study to evaluate the baseline asthma knowledge, attitude, and practice of primary caregivers of asthmatic children [8]. The combined instrument (i.e., AKQ and CARAT) was reviewed in the Jiann-Ping Hsu College of Public Health for face and content validity.

***Measures related to the questionnaire:*** The questionnaire included the following measures to which the responses were recorded as a binary response (yes/no) or as a 5-point Likert-scale response: “strongly disagree (SD = 5)”, “disagree (D = 4)”, “neither agree, nor disagree” (N = 3), “agree” (A = 2), and “strongly agree” (SA = 1), respectively. This implies that the correct response was either “strongly agree” or “strongly disagree” and ultimately dependent on the correct response for each specific research question. The survey instrument for this study was divided into six sections:

- Demographic characteristics include the child’s sex, age, and parental SES (i.e., education and income levels) (Questions 1–5).
- Background of the study population and health characteristics including family history, access to healthcare, and its utilization (Questions 6–17).
- Perceived susceptibility and severity of asthma through research questions on asthma-related knowledge and understanding of risk factors. This has been subcategorized into three parts: Section 3a: Myths and beliefs regarding asthma (Question 18–24); Section 3b: Level of knowledge about the disease (Question 25–30); and Section 3c: Knowledge about associated aspects of asthma (Question 34–41).
- Environmental exposures (Questions 34–41).
- Perceived barriers to and benefits of practicing proper asthma-management behaviors, including those regarding costs, time, and access to healthcare facilities. The research questions about the practice of caregivers in managing asthma in their children are from Questions 42–52.
- The psychosocial and emotional burden of asthma on the child and the caregiver (Question 53–55).

The asthma knowledge score was estimated as follows:

The first category was “myths and beliefs regarding asthma” (Q18–24) which contained seven questions where each question had a score of 1–5. The possible total score of 35 was subcategorized as follows: poor (score 7–14), moderate (score 15–21), or good (score 22–35).

The second category was “level of knowledge about the disease” (Q25–30) which contained six questions where each question had a score of 1–5. The possible total score of 30 was subcategorized as follows: poor (score 6–12), moderate (score 13–18), or good (score 19–30).

The third category was “knowledge about associated aspects of asthma” (Q31–33) which contained three questions where each question had a score of 1–5. The possible total score of 15 was subcategorized as follows: poor (score 3–6), moderate (score 6–9), or good (score 10–15). Missing answers to the asthma knowledge questionnaire were scored as ‘0′.

The total sum of all three categories was calculated to be 80, with higher scores indicating greater knowledge of asthma, and this total was subcategorized into three levels: poor (score 20–40), moderate (score 41–60), or good knowledge (score 61–80). In other words, those respondents who obtained an asthma knowledge score above 61 were considered as having high-level knowledge, while the scores between 41 and 60 were considered to have medium-level knowledge. A score below 40 was considered to represent low-level knowledge. The other segments contained closed-ended questions about the practice of caregivers in managing asthma in their children, environmental exposures, and the psychosocial and emotional burden of asthma on the child and the caregiver. The questionnaire was translated into the local language (i.e., Yoruba) and reviewed by a local research expert. A pilot study was conducted among 10 caregivers of children with asthma to ensure that the language and content were appropriate and to ensure the overall clarity and suitability of the instrument.

### 2.3. Data Analysis

Data were entered into the IBM Statistical Package for Social Science (SPSS) for Windows, version 25.0 [11]. Descriptive results were compiled by computing frequencies for categorical variables. The continuous variables were summarized using means and SDs for normally distributed variables. Regression analyses were performed to test whether there were significant associated factors affecting the outcomes. The Pearson correlation coefficient (r) was used to measure the strength and direction of the relationship between variables. The Chi-square test was used to test for categorical variables’ association. Statistical significance was claimed at *p* ≤ 0.05, and we also provide the 95% CI.

## 3. Results

A total of 178 caregivers completed the interviewer-administered questionnaire, with a response rate of 98.9%. Table 1 summarizes the demographic and background characteristics of the study participants for both caregivers and their children. Of the 178 caregivers of children with asthma, 156 (87.6%) were mothers, 11 (6.2%) were fathers, and 10 (5.6%) were grandparents, with the rest being guardians and relatives. The mean age of the children of the caregivers was 3.0 (±1.01) years, ranging from 1 to 5 years; 51.7% of them were male, and 48.7% were female. Most of the respondents (58.3%) had low levels of education (i.e., no education, attended primary and/or secondary school), whereas 34.8% (n = 62) of the respondents attended college but did not receive a degree, and 6.7 percent of the respondents said they had completed a college degree. The participants received most of the information about asthma from primary care doctors/nurses, relatives/friends, and the Internet (68%, 12.4%, and 2.8%, respectively).

Table 2 shows the Chi-square analysis result, which indicates that educational status in caregivers of children with asthma had no association with asthma control. However, there is an association between the income of caregivers and asthma control. The greater proportion of the caregivers of children with poorly controlled asthma had a low education and a monthly income of less than the United States Dollar (208 (78.1%)). Income and asthma control are not independent of each other and there is a statistically significant relationship between these variables.

Table 3 and Table 4 present the analysis of the caregiver’s responses to the asthma knowledge questions. The total calculation (n) and analysis for section (See Table 3) and bivariate analysis (See Table 4) were conducted for 178 participants, and this was the exact number of responses from this study. The mean score for the first category in the asthma knowledge questions (myths and beliefs regarding asthma) was 22.81 (±3.62) out of 35. Most of the respondents (66.9%) had a good knowledge score, 31.5% had a sufficient knowledge score, and only 1.7% had poor knowledge in this category.

The mean score for the second category (general knowledge about asthma) was 24.84 (±3.56) out of 30. Most of the respondents (99.9%) had high knowledge scores with no poor knowledge in this category.

The mean score for the third category (knowledge about associated aspects of asthma) was 11.83 (±1.90) out of 15. Most of the respondents (94.4%) had good knowledge, 4.5% had sufficient knowledge, and 1.1% had poor knowledge in this category. The mean value for the total knowledge score was 59.48 (±4.78) out of 80. Overall, more than half of the respondents (58.4%) had moderate knowledge and 41.6% had sufficient knowledge of asthma, misperceptions, and associated aspects of asthma.

Table 5 summarizes the risk-perception level of caregivers toward asthma in their children. The majority of the respondents, 78.7% (140), had a moderate perceived susceptibility score of between 3 and 5 (*X*^–^ = 3.66, SD = 1.191). Most of the respondents believed their children were susceptible to risk and severity of asthma based on their family history (40.4%), exposure to indoor pollutants and allergens linked to asthma such as type of cooking fuel (98.9%), mold/mildew (32%), pets (27%), and other environmental factors.

Table 6 summarizes the relationships between the asthma-perception level and knowledge of asthma triggers of the participants. Univariate analysis was performed to investigate associations between the dependent variable of perceived susceptibility to an asthma attack and five independent variables associated with asthma triggers. A statistically significant association was found between the level of perceived susceptibility of an attack and the asthma triggers variable (allergens (χ2 = 0.0259) and generated smoke only (χ2 = 0.0352). No statistically significant association was found between the caregiver’s perception level and other asthma triggers (URI, weather, and exercise variables).

Table 7 shows the independent predictors of allergen-specific exposure. Binary logistic regression analysis was performed to identify predictors of allergen-specific exposure. The majority of the caregivers believe that their child’s asthma is triggered by exposure to dust (OR = 0.324; 95% CI = 10.131–0.803; *p* = 0.015) and cleaning supplies (OR = 0.244; 95% CI = 0.88–0.677; *p* = 0.007), as these predictors were statistically significant.

Table 8 summarizes the relationships between the caregiver’s pre-identification of early childhood asthma symptoms (dry cough at night and wheezing) and clinic visits. Caregivers with knowledge of wheezing (“yes” category) were less likely to take their child to the clinic for asthma symptoms, a statistically significant result (*p* = 0.001); however, they were more likely to visit the clinic for asthma symptoms (*p* = 0.000).

Table 9 summarizes the relationships between the caregiver’s pre-identification of early childhood asthma symptoms (dry cough at night and wheezing) and hospital admissions. Asthma-related hospitalization in children within the last 12 months was significantly associated with the caregiver’s knowledge about childhood asthma symptoms, e.g., dry cough (*p* = 0.020).

Table 10 summarizes the relationships between the caregiver’s pre-identification of early childhood asthma symptoms (dry cough at night and wheezing) and administering asthma medication. Caregivers with knowledge of wheezing are more likely to administer medicine for quick relief of asthma symptoms, a statistically significant result, *p* = 0.0005; however, those with the knowledge of a dry cough were less likely to administer medicine for quick relief of asthma symptoms, a statistically significant result, *p* = 0.003. Interestingly, the odds for knowledge of wheezing were twice that of a dry cough (1.313,1.086) and were statistically significant.

Table 11 and Table 12 present the tabulation of the asthma knowledge, education, and income level of participants and their practice in managing their child’s asthma. No significant correlation was found between asthma-management practices and the asthma knowledge of caregivers, χ2 (1) = 0.026, *p* = 0.872. In total, 46 (64.8%) participants with an educational attainment of secondary school and below engaged in poor asthma-management practices, compared to 25 (35.2%) with some college and college graduates; the difference was not significant. Caregivers with an income of or above NGN 75,000 (USD 208) were 3.0 times more likely to practice proper asthma behavior than those with a lesser income.

## 4. Discussion

In our study, research participants were interviewed during their visit to the child welfare clinic at the primary healthcare centers (PHCs) and pediatric department of the state hospital where patients with unstable, difficult-to-control/severe asthma are received; therefore, participants’ level of knowledge and practice may vary for several reasons. Parents whose children have difficult or hard-to-control asthma will be more likely to visit the PHCs and state hospitals, visit the doctors/nurses often, and as a result be more knowledgeable about asthma. Another reason may be that the state clinic has specialized pediatric doctors who provide specialized care and education to patients each time they visit the hospital. However, in this analysis, study participants were not subcategorized according to the healthcare settings; hence, the caregiver’s level of knowledge and practice is not determined by the level of care received (whether caregivers attended the CWC at the PHC/state hospital or not).

One potential factor that may explain the utilization of PHCs and the pediatric department of the state hospital may be the caregiver’s perception of disease severity and control. The results of our study suggest that the majority of the caregivers were aware of their child’s asthma status and reported the severity of their child’s asthma based on their perceptions. In addition, it is important to take into consideration the fact that the assessment of childhood asthma severity is based mainly on the caregiver’s personal reports and not necessarily a medically diagnosed report. Additionally, we found that most of the cases of severe asthma in children are a result of caregivers’ poor perception of asthma symptoms in their children and difficulties in caregivers’ recognition of signs and symptoms of the disease in their children. While the caregiver’s perception of the disease has been linked to poor asthma management, in this study, there were other factors such as the degree of a child’s exposure to pollutants and level of asthma severity that contributed to the caregiver’s asthma-care-seeking behavior and treatment for their children. Few previous studies have reported on caregivers’ perception of asthma severity levels (i.e., intermittent, mild persistent, moderate persistent, and severe persistent levels) in children and the associated level classification of asthma-control measures (i.e., well-controlled, not well-controlled, and very poorly controlled) [2,12,13].

The current study shows that the majority of the respondents reported no family history of asthma, and the findings differ from prior studies that reported a positive asthma history among most of their respondents [2,12]. Previous clinical studies showed that the family history was associated with an increased number of asthmatic children that attended the pediatric chest clinic of the Wesley Guild Hospital (WGH), Ilesha, Nigeria [2]. According to AlOtaibi and AlAteeq (2018), most of the parents and guardians of children with asthma that attended the general pediatric and pediatric pulmonology outpatient clinics at King Abdullah Specialist Children’s Hospital, King Abdulaziz Medical City for the National Guard, Riyadh, Saudi Arabia, reported a family history of asthma and allergy [12]. The relatively low response to family history reported in the current study may be because family history is often overlooked as an important risk factor and caregivers may not consider family history as an indicator of risk for childhood asthma due to poor knowledge, myths, misconceptions, or cultural factors.

Further, since the source of asthma information is known to have an impact on knowledge and asthma-management practices, it may be worthwhile to discuss caregivers’ sources of health information. The Internet is a growing source of health information in developing countries such as Nigeria; however, in this study, 68% of participants reported primary care providers such as doctors and community health nurses as their source of information about asthma. Considering the poor standard of asthma care and the lack of asthma educators in many health facilities in Nigeria, the roles of the radio, TV, and Internet as alternative sources of asthma education need to be enhanced. Professional and certified health education communication channels on asthma-guidelines-based care and asthma self-management education need to be available for patients and their families.

Most of the participants in this study had a moderate knowledge score in the total knowledge and all knowledge subcategories (myths and beliefs, general knowledge, and knowledge of associated aspects) using the validated AKQ. A local study investigating a similar topic reported that the level of knowledge about childhood asthma was significantly poor among caregivers (38.5%) in the pediatric chest clinic of the Wesley Guild Hospital (WGH), Ilesha, Nigeria, using a different AKQ with a mean knowledge score of 11.2 (SD = 3.7) which ranged from 1 to 18 [5]. The difference in their findings and this study may be related to the lower educational qualifications of caregivers in Ilesha (a smaller city) compared to Ibadan (a metropolitan city).

In the current study, participants were the most knowledgeable about asthma pathophysiology (such as the role of airway inflammation), triggers of asthma exacerbation in their children, and questions related to diagnosis and treatment. The findings agree with the reports from Rastogi et al., 2013, and AlOtaibi and AlAteeq, 2018 [8,10,14]. These studies found a better knowledge among caregivers in questions related to symptoms of asthma, asthma triggers in their children, diagnosis and treatment, pathogenesis, and the nature of the disease. The findings of this study agree with Rastogi et al., 2013 [8], who found that caregivers had a better knowledge of asthma triggers and were aware of the importance of using controller medications. Additionally, our study showed that caregivers were knowledgeable about the associated aspects of asthma, with 94.9% having a moderate level of knowledge. However, a severe deficit of knowledge was observed particularly in questions related to the myths and beliefs about asthma, where 31.5% of participants had fair knowledge and 1.7% had poor knowledge. In this subcategory of knowledge (myths and beliefs regarding asthma), caregivers were presented with statements such as “asthma is a curse from the gods,” “childhood asthma can be cured by homemade or herbal remedies,” and “pray before going to the clinic when your child has an asthma attack.” Caregivers’ misconceptions about asthma medication are reflected in their actual practice in managing asthma in their children, as almost half of the respondents in this study reported providing alternative therapy for asthma such as massage, homemade, and herbal remedies. Such therapies were more widely used by caregivers whose knowledge score was relatively low. This is consistent with the findings from previous international studies [2,14,15,16].

One potential factor that may explain the caregivers’ perceived susceptibility to risk and severity of asthma may be the underlying knowledge of asthma triggers. Factors associated with the asthma-perception level among caregivers were a family history of asthma; indoor environmental allergens, e.g., house dust, cooking fuel, pets, mold, and other environmental pollutants such as tobacco smoke. Exposure to allergens may likely have contributed to asthma severity as reported by caregivers of children with asthma. The outcome of this study showed that caregiver’s knowledge of allergens and generated smoke was significantly associated with perceived susceptibility to an asthma attack, which was similarly reported by Rastogi et al., 2013, who found that knowledge of perceived triggers (e.g., infection, type of food consumed, and exercise) influenced caregiver’s perception of an asthma attack [8]. Given the level of awareness about common triggers of asthma exacerbation, caregivers’ perception of risk can be low in terms of the frequency of symptoms and decreased risk of severe asthma attacks. The avoidance of exposure to dust and cleaning supplies in children with asthma will go a long way in ensuring good asthma control and ultimately improving their quality of life.

The current study showed that the severity of asthma was also associated with caregivers’ knowledge of early asthma symptoms. Caregivers with knowledge of early asthma symptoms (i.e., recurrent wheezing and dry cough at night) reported a high usage of quick-relief medicine for their children during asthma exacerbations. Similarly, the frequency of asthma attacks was found to be significantly related to the pre-identification of wheezing and a dry cough at night as early symptoms of childhood asthma. In this study, caregivers with knowledge of early wheezing episodes (especially during or after exercise) were less likely to visit the clinic but more likely to visit the clinic with recurring dry cough episodes. The results suggest that caregivers would prefer to use cough medicines purchased from the patent and proprietary medicine vendors (PPMVs) in their community or use home remedies to help relieve cough symptoms in their children. These findings were similar to those reported by Rastogi et al. (2013) and Soo and Tan (2014) [8,15]. Despite the relatively good knowledge of asthma in this study, caregivers did not visit the primary health care center for asthma exacerbations such as wheezing but went to emergency visits for night-dry cough, which led to hospital admissions. This also implies that caregivers are unable to translate their knowledge and awareness into action in situations of asthma exacerbations. Poor knowledge of early asthma symptoms may be associated with either frequent hospital admission or low clinic visits (i.e., frequency of healthcare-seeking behavior).

The current study shows that socio-demographic class is an important predictor of asthma control, which has also been reported in other studies [2,17,18]. In these studies, the authors found that caregivers’ low income and unemployment status were significant predictors of poor asthma control in Pakistan, Nigeria, and the U.S.A. In this current study, the result showed that the level of asthma control was not significantly related to caregivers’ education, although a higher proportion of caregivers with children having well-controlled asthma had at least a secondary level of education. This observation may be related to the fact that highly educated caregivers may have inadequate knowledge, incorrect caregiver-reported diagnosis, and wrong perception of asthma-management practices [2,13,14,19]. On the other hand, income level was significantly associated with asthma control in this study. Caregivers in the low-income category face the risk of a financial burden that makes them unable to afford the cost of proper asthma care for their children, hence predisposing them to poor management of childhood asthma [18]. This also implies that if asthma treatment services are easily accessible and affordable to all socio-economic classes in the community, asthma exacerbations will be reduced, leading to better asthma practices and an improved quality of life for both the caregivers and their children.

In summary, our results suggest that caregivers of asthmatic children have a moderate knowledge of the common asthma symptoms and aggravating factors but poor knowledge regarding fundamental asthma treatment and management. The challenge for future health promotion and public health lies in the careful identification of caregivers’ experiences, their needs in managing the care of their asthmatic child, and factors that facilitate or hinder the performance of appropriate asthma-management-practice behaviors. This study revealed that caregivers, particularly parents, play an important role in shaping healthcare behavior in their children. For better asthma management and control, an optimal public health approach is needed to identify and target underserved communities that suffer poorer asthma outcomes and to improve caregivers’ knowledge and skills in asthma-management practices [20,21,22]. In order to holistically improve child asthma care and outcomes, asthma-education campaigns including community-targeted initiatives to engage caregivers, healthcare professionals, and the community at large are recommended [22,23]. This study, however, contributes new findings to the existing literature conducted locally about childhood asthma-management practices in Nigeria and how social and environmental factors influence the proper asthma-management practice behaviors of caregivers.

The findings in this report are subject to a few limitations. First, this study was carried out in primary health care centers and state pediatric outpatient clinics with the selection of participants through a convenience sampling method. Although data saturation was achieved in the analysis, there is a possibility that other important aspects of the caregiver’s experience of asthma management were not captured. Secondly, we used an interviewer-administered questionnaire in this research; interviewer bias recall bias cannot be excluded. The information on the frequency and severity of asthma symptoms reported by caregivers may not be accurate. Study participants may erroneously provide inaccurate or false reports depending on their ability to recall past asthma episodes, hence there is a recall bias. Despite these limitations, this study has contributed to the inadequate data on childhood asthma and factors associated with proper asthma management in a developing country. This study has also revealed insights into the level of knowledge, attitude, and practice behaviors of caregivers in the management of asthma in their children in developing countries such as Nigeria, as well as having examined factors associated with the barriers and benefits of proper asthma-management practices.

## 5. Conclusions

In conclusion, this study shows the caregiver’s perception and understanding of asthma, as well as the benefits and barriers of successful asthma-management practices (i.e., the decision of asthma care and adherence to appropriate asthma preventive and management measures). Understanding caregivers’ experiences of childhood asthma management can play a pivotal role in the effort to achieve a sustainable approach to improving care for children with asthma. Given the limited healthcare resources in rural Nigeria, the innovative use of multimedia platforms and help from other allied health professionals such as community health extension workers (CHEWS) and patent medicine vendors (PMVs) should be considered for the delivery of asthma education.

## Figures and Tables

**Table 1:** Demographics and background characteristics of the participants.

Demographic Data (n = 178)	*N*	%
**Education Level of Caregivers**		
No Education	6	3.4
Primary School	23	12.9
Secondary School	75	42.1
College	62	34.8
College Graduates	12	6.7
**Relationship to Child**		
Mother	156	87.6
Father	11	6.2
Grandparents	10	5.6
Other Relatives or Guardians	1	0.6
Family Income (Monthly)		
0–75,000	106	59.6
>75,00–100,000	72	40.4
>100,000	0	0
Gender of Child		
Male	92	51.7
Female	86	48.3
**Sources of Information about Asthma**		
	**N (n = 178)**	**%**
Primary Care Provider (i.e., Doctor, Nurse)	121	68
Internet/social media	5	2.8
Relatives/Friends	22	12.4
Other Sources (Radio/TV)	30	16.9

**Table 2:** Descriptive statistics based on asthma control.

Variable	Poorly Controlled		Well-Controlled		*p*-Value
	N	%	N	%	
**Education**					**0.329**
Secondary School and Below	19	59.4	85	58.2	
Some College and College Graduate	13	40.6	61	41.8	
**Income**					**0.018**
<NGN 75,000 (USD 208)	25	78.1	81	55.5	
>NGN 75,000 (USD 277)	7	21.9	65	44.5	

Bold represents a *p*-value, and hypothesis tests results with statistical significance.

**Table 3:** Asthma knowledge questions.

	Strongly Agree	Agree	Neither Agree/Nor Disagree	Disagree	Strongly Disagree
	N (%)	N (%)	N (%)	N (%)	N (%)
**Section 3: Myths and beliefs regarding asthma.**					
Asthma is a curse from the gods.	90 (50.6)	48 (27.0)	34 (19.1)	4 (2.2)	2 (1.1)
Childhood asthma can be cured by traditional herbs/ homemade or herbal remedies.	20 (11.2)	24 (13.5)	60 (33.7)	58 (32.6)	16 (9.0)
Your child may outgrow asthma.	6 (3.4)	16 (9.0)	25 (14.0)	89 (50.0)	42 (23.6)
Inhaler use can lead to dependence or addiction.	4 (2.2)	33 (18.5)	44 (24.7)	67 (37.6)	30 (16.9)
After a child’s asthma attack, once the coughing is over, the use of an inhaler or medications should be stopped?	12 (6.7)	27 (15.2)	21 (11.8)	89 (50.0)	29 (16.3)
It is not good for children to use an inhaler for too long.	4 (2.2)	22 (12.4)	50 (28.1)	63 (35.4)	39 (21.9)
When a child has an asthma attack, it is best to pray before going to the emergency room/clinic especially when symptoms are mild.	19 (10.7)	26 (14.6)	30 (16.9)	63 (35.4)	40 (22.5)
**Section 3: Level of Knowledge about Asthma**					
The main cause of asthma is airway inflammation.	3 (1.7)	3 (1.7)	29 (16.3)	65 (36.5)	78 (43.8)
Asthma attacks can be prevented if medications are taken even when there are no symptoms between attacks.	4 (2.2)	11 (6.2)	18 (10.1)	77 (43.3)	68 (38.2)
Flu (common cold and cough) infections are the main causes or triggers of asthma attacks.	5 (2.8)	8 (4.5)	17 (9.6)	59 (33.1)	89 (50.0)
If an asthmatic child gets the flu, you should apply the inhaler even if there is no coughing or wheezing	5 (2.8)	20 (11.2)	31 (17.4)	61 (34.3)	61 (34.3)
Asthmatic children might have attacks that are severe enough to require hospitalization in an intensive care unit or they might even die from an attack.	1 (0.6)	2 (1.1)	5 (2.8)	45 (25.3)	125 (70.2)
Some medications for asthma do not work unless they are administered every day.	4 (2.2)	14 (7.9)	47 (26.6)	55 (30.9)	58 (32.6)
**Section 3: Knowledge about an associated aspect of asthma**					
Children who have asthma should not participate in sports that make them run too much.	3 (1.7)	5 (2.8)	11 (6.2)	61 (34.3)	98 (55.1)
It is best not to smoke or let anyone else smoke near a child who has asthma.	1 (0.6)	2 (1.1)	1 (0.6)	40 (22.5)	134 (75.3)
If the parents/guardians of a child with asthma smoke outside the house, it will not affect the child.	48 (27.0)	47 (26.4)	19 (10.7)	31 (17.4)	33 (18.5)

**Table 4:** Score of asthma knowledge.

Scores of Categories						
n = 178	Good Knowledge		Fair Knowledge		Poor Knowledge	
	N	%	N	%	N	%
Category 1: Myths and beliefs regarding asthma	22–35		15–21		7–14	
	119	66.9	56	31.5	3	1.7
Category 2: Level of Knowledge about Asthma	19–30		13–18		6–12	
	169	94.9	9	5.1	0	0
Category 3: Knowledge about an associated aspect of asthma	10–15		7–9		3–6	
	168	94.4	8	4.5	2	1.1

**Table 5:** Summary score of perceived susceptibility level of the respondents.

Perceived Susceptibility Summary Score							
**Variable**	Highly Susceptible		Moderately Susceptible		Little/No Susceptibility		*X*^–^ ± SD
Score of Categories	6–8		3–5		1–2		
	**N**	**%**	**N**	**%**	**N**	**%**	**3.66 ± 1.191**
	14	7.9	140	78.7	24	13.5	

**Table 6:** Association between asthma perception level and knowledge of asthma triggers.

Variables	Perception Level (Number of Caregivers)			Mean	95% CI	*p*-Value
	Low Susceptibility (3)	Moderate Susceptibility (2)	High Susceptibility (1)			
URI	8	19	2	3.34	2.84, 3.84	0.14
Weather	20	117	10	3.63	3.45, 3.82	0.53
Exercise	6	35	6	3.77	3.38, 4.14	0.54
Allergens	20	83	8	3.52	3.30, 3.74	**0.035**
Generated Smoke	1	7	7	4.73	3.74, 5.72	**0.0259**

Note: Bold represents a *p*-value, and hypothesis tests results with statistical significance.

**Table 7:** Estimated prevalence and odds ratio for independent predictors of allergen-specific exposure.

Predictor	*B*	SE	OR (95%CI)	*p*-Value	*R* ^2^
Dust	−1.12	0.46	0.324 (0.131,0.803)	**0.015**	Nagelkerke *R*^2^ **13.2%**
Cleaning Supplies	−1.41	0.52	0.244 (0.88,0.677)	**0.007**	

**Key:** *B* = coefficient of regression, SE = standard error, *R*^2^ = Nagelkerke *R*^2^ value, Bold represents a *p*-value, and hypothesis tests results with statistical significance.

**Table 8:** Poisson regression analysis of early symptoms of asthma (dry cough at night and wheezing) and clinic visits, associated with the caregiver’s pre-identification of early childhood asthma symptoms and frequency of asthma attacks.

Predictor	Estimates	Std. Error	IRR (95%CI)	*p*-Value
Wheezy chest during or after physical activity	0.500	0.1532	0.607 (0.449,0.819)	**0.001**
Dry cough at night	0.776	0.2189	2.173 (1.415,3.337)	**0.000**

Note: Bold represents a *p*-value, and hypothesis tests results with statistical significance.

**Table 9:** Poisson regression analysis of early symptoms of asthma (dry cough at night and wheezing) and hospital admissions, associated with the caregiver’s pre-identification of early childhood asthma symptoms and frequency of asthma attacks.

Predictor	Estimates	SE	IRR (95%CI)	*p*-Value
Wheezy chest during or after physical activity	−0.339	0.25	0.712 (0.434,1.169)	0.17
Dry cough at night	0.969	0.41	2.636 (1.163,5.973)	**0.020**

**Key:** IRR = incidence rate ratio, SE = standard error, df = 1, Bold represents a *p*-value, and hypothesis tests results with statistical significance.

**Table 10:** Poisson regression analysis of early symptoms of asthma (dry cough at night and wheezing) and administering asthma medication, associated with caregiver’s pre-identification of early childhood asthma symptoms and severity of asthma attacks.

	Estimates	Std. Error	IRR (95%CI)	*p*-Value
Wheezing	0.273	0.09	1.313 (1.313,1.086)	**0.000**
Dry Cough	−0.336	0.11	0.715 (0.572,0.894)	**0.003**

Note: Bold represents a *p*-value, and hypothesis tests results with statistical significance.

**Table 11:** Association between asthma knowledge level and management-practice scores of respondents.

Variables	Asthma Management Practice Level		*p*-Value
	Poor Practices	Good Practices	
**Asthma Knowledge Level**	(n %)		
Fair	42 (59.2)	62(57.9)	0.872
Good	29 (40.8)	45(42.1)	
**Education level**			
Secondary School and Below	46 (64.8)	58 (54.2)	0.689
Some Colleges and College Graduate	25 (35.2)	49 (45.8)	
**Income level**			
<NGN 75,000 (USD 208)	53 (74.6)	53 (49.5)	**0.001**
>NGN 75,000 (USD 277)	18 (25.4)	54 (50.5)	

Note: Bold represents a *p*-value, and hypothesis tests results with statistical significance.

**Table 12:** Results of binary logistic regression analysis for odds of practicing asthma-management behavior against the predictor variable (income).

Predictor Variable	*B*	SE	Nagelkerke *R*^2^	OR (95%CI)	*p*-Value	Tests of Model Coefficient
Income	1.099	0.334	**8.5%**	3 (1.558,5.778)	**0.001**	11.506

Note: Bold represents a *p*-value, and hypothesis tests results with statistical significance.

## Data Availability

Data are unavailable due to privacy.
